# 2,5-Bis(1,3-dithiol-2-yl­idene)-1,3-dithiol­ane-4-thione

**DOI:** 10.1107/S1600536811051518

**Published:** 2011-12-10

**Authors:** Kazumasa Ueda, Kenta Suzuki, Kei Kunimoto, Kenji Yoza

**Affiliations:** a Division of Basic Engineering, Faculty of Engineering, Shizuoka University, Johoku 3-5-1, Hamamatsu, Shizuoka 432-8561, Japan; b Department of Materials Science and Chemical Engineering, Graduate School of Engineering, Shizuoka University, Johoku 3-5-1, Hamamatsu, Shizuoka 432-8561, Japan; c Bruker AXS Co. Ltd, Moriya-cho 3-9, Kanagawa-ku, Kanagawa, Kanagawa 221-0022, Japan

## Abstract

The asymmetric unit of the title compound, C_9_H_4_S_7_, contains two independent mol­ecules, in one of which the central five-membered ring is disordered over two orientations in a 0.924 (3):0.076 (3) ratio. The mol­ecular skeleton is almost planar: the average distance of the atoms from their mean plane is 0.128 (7) Å in the ordered mol­ecule, and 0.088 (5) and 0.123 (2) Å in the major and minor disorder components, respectively. The ordered and disordered mol­ecules form separate columns by stacking along the *b* axis. Adjacent columns inter­act *via* short S⋯S [3.33 (2), 3.434 (3), 3.444 (2), 3.503 (2), 3.519 (3) and 3.53 (4) Å] and S⋯H [2.814 (2), 2.87 (7), 2.92 (2), 2.9269 (18), 2.93 (2), 2.94 (2), 2.939 (2), 2.967 (2) and 2.974 (1) Å] contacts.

## Related literature

For background to 2,5-di(1,3-dithiol-2-yl­idene)-1,3-dithiol­ane-4-thione derivatives, see: Iwamatsu *et al.* (1999 ▶, 2000 ▶); Wang *et al.* (2005 ▶, 2007 ▶); Hiraoka *et al.* (2005 ▶); Fujiwara *et al.* (2006 ▶, 2007 ▶); Ueda & Yoza (2009*a* ▶,*b* ▶,*c* ▶). For the synthesis, see: Ueda *et al.* (2010 ▶). For van der Waals radii, see: Bondi (1964 ▶).
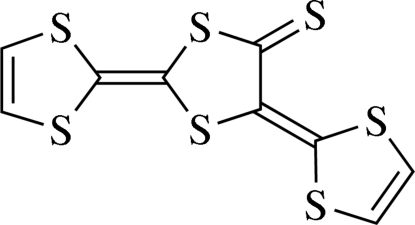


## Experimental

### 

#### Crystal data

C_9_H_4_S_7_
*M* *_r_* = 336.54
Monoclinic, 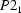

*a* = 17.669 (5) Å
*b* = 3.9110 (11) Å
*c* = 18.380 (5) Å
β = 108.177 (4)°
*V* = 1206.8 (6) Å^3^
*Z* = 4
Mo *K*α radiation
μ = 1.27 mm^−1^
*T* = 93 K
0.09 × 0.02 × 0.02 mm

#### Data collection

Bruker APEXII CCD area-detector diffractometer
Absorption correction: multi-scan (*SADABS*; Sheldrick, 1996 ▶) *T*_min_ = 0.894, *T*_max_ = 0.975
6965 measured reflections
4975 independent reflections
3396 reflections with *I* > 2σ(*I*)
*R*_int_ = 0.054

#### Refinement

*R*[*F* ^2^ > 2σ(*F* ^2^)] = 0.061
*wR*(*F* ^2^) = 0.107
*S* = 0.98
4975 reflections
344 parameters
421 restraints
H-atom parameters constrained
Δρ_max_ = 0.63 e Å^−3^
Δρ_min_ = −0.52 e Å^−3^
Absolute structure: Flack (1983 ▶), 1849 Friedel pairs
Flack parameter: −0.18 (18)

### 

Data collection: *APEX2* (Bruker, 2010 ▶); cell refinement: *SAINT* (Bruker, 2010 ▶); data reduction: *SAINT*; program(s) used to solve structure: *SHELXS97* (Sheldrick, 2008 ▶); program(s) used to refine structure: *SHELXL97* (Sheldrick, 2008 ▶); molecular graphics: *SHELXTL* (Sheldrick, 2008 ▶); software used to prepare material for publication: *XCIF* (Bruker, 2010 ▶).

## Supplementary Material

Crystal structure: contains datablock(s) I, global. DOI: 10.1107/S1600536811051518/fy2030sup1.cif

Structure factors: contains datablock(s) I. DOI: 10.1107/S1600536811051518/fy2030Isup2.hkl

Supplementary material file. DOI: 10.1107/S1600536811051518/fy2030Isup3.cml

Additional supplementary materials:  crystallographic information; 3D view; checkCIF report

## Figures and Tables

**Fig. 1.**
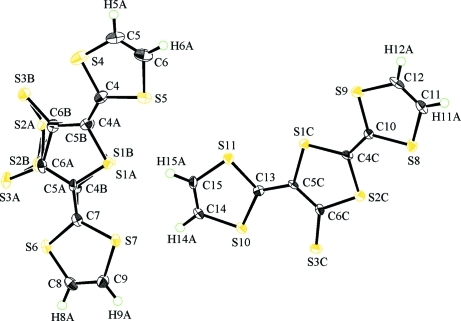
The molecular structure of (I) showing atom labeling and 50% probability displacement ellipsoids for non-H-atoms.

**Fig. 2.**
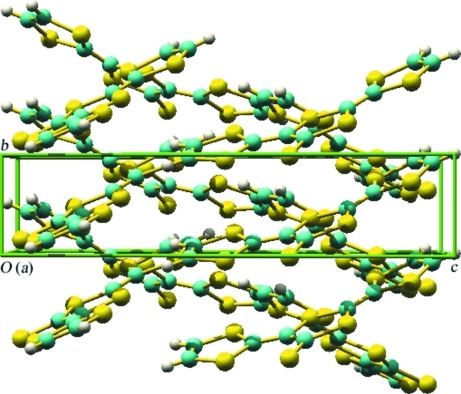
Projection of the crystal packing of (I) down the bc plane.

**Fig. 3.**
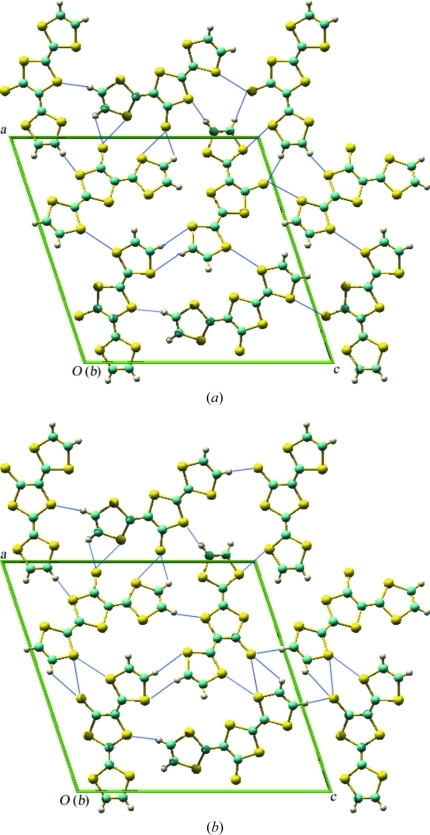
(a) Projection of the crystal packing of molecules A and C in (I) down the ac plane. The S···S and S···H contacts are shown with blue solid lines. (b) Projection of the crystal packing of molecules B and C in (I) down the ac plane. The S···S and S···H contacts are shown with blue solid lines.

## supplementary crystallographic information

### Comment

Donor molecules featuring a skeleton of 2,5-di(1,3-dithiol-2-ylidene)-1,3-dithiolane-4-thione (I) and 2,5-di(1,3-dithiol-2-ylidene)-1,3-dithiolane-4-quinone (II) are used for the preparation of charge transfer (CT) complexes with magnetic metal anions (Wang *et al.*, 2005, 2007; Hiraoka *et al.*, 2005; Fujiwara *et al.*, 2006, 2007). In CT salts these molecules can form unique crystal structures with channels in addition to the usual layer stacking structures as a result of intermolecular S···S contacts. Through an investigation of the unique molecular arrangements of the derivatives of (I) and (II) in their crystals, we found that the derivatives of (I) are stacked in the same orientation and the derivatives of (II) are alternately stacked in opposite directions (Ueda & Yoza, 2009*a*, 2009*b*, 2009*c*; Ueda *et al.*, 2010). This result suggests that the stacking orientation of the derivatives is largely influenced by their molecular skeletons. To identify the stacking orientation of skeleton (I), we synthesized (I) and investigated its crystal structure.

The asymmetric unit contains two crystallographically independent molecules. One of the two independent molecules shows orientational disorder in the C=S containing five-membered ring with occupancies of 0.924 (3) (molecule A) and 0.076 (3) (molecule B). The other molecule of the asymmetric unit, molecule C, is ordered. The framework of (I) is almost planar: the mean deviation of the atoms from their mean plane is 0.088 (5) Å for A, 0.123 (2) Å for B and 0.128 (7) Å for C.

In the crystal structure, two different orientations of the molecules are present. Both orientations enclose a dihedral angle of about 27° with the *ac* plane, but the mean plane of the molecules is nearly parallel either with (011) or with (01–1) (Fig. 2). Molecules with the same orientation form stacks along the *b* axis. The stacked molecules are separated by interplanar distances greater than 3.54 Å and have a fairly poor overlap. However some effective side-by-side contacts are observed between molecules of adjacent columns (Fig. 3). The interaction between adjacent columns is accomplished through contacts between different sulfur atoms: S9···S3B^i^ = 3.33 (2) Å [(i): 1–*x*, 1/2+*y*, 1–*z*]; S8···S3A^ii^ = 3.434 (3) Å [(ii): *x*, 1+*y*, 1+*z*]; S6···S6^iii^ = 3.444 (2) Å [(iii):–*x*, 1/2+*y*, –*z*]; S3C···S10^i^ = 3.503 (2) Å; S4···S9^i^ = 3.519 (3) Å; S2B···S3A^iv^ = 3.53 (4) Å [(iv): *x*, 1+*y*, *z*]; and through contacts between sulfur and hydrogen atoms: S2C···H9A^i^ = 2.814 (2) Å, S1B···H15A = 2.87 (7); S3B···H11A^iv^ = 2.92 (2) Å; S5···H6A^i^ = 2.9269 (18) Å; S3B···H11A^v^ = 2.93 (2) Å [(v): *x*, *y*, *z*–1]; S3B···H12A^vi^ = 2.94 (2) Å [(vi): 1–*x*, –1/2+*y*, 1–*z*]; S2C···H9A*^vii^* = 2.939 (2)Å [(vii): –*x*, –1/2+*y*, 1–*z*]; S3A···H8A^iii^ = 2.967 (2) Å; S3C···H14A^viii^ = 2.974 (1) Å [(viii): –*x*, 1/2+*y*, 1–*z*]. These distances are shorter than the sum of corresponding van der Waals radii, *i.e.*, 3.60 Å for S···S and 3.00 Å for S···H (Bondi, 1964).

### Experimental

Compound (I) was synthesized by a modification of the method used for the preparation of 2-[4,5-bis(ethylsulfanyl)-1,3-dithiol-2-ylidene]-5-(4,5-diiodo-1,3-dithiol-2-ylidene)-1,3-dithiolan-4-thione (Ueda *et al.*, 2010). Bis(tetra-*n*-butylammonium)bis[2-(1,3-dithiol-2-ylidene)-1,3-dithiole-4,5-bis(thiolate)]zinc (194 mg, 0.170 mmol) reacted with 2-methylsulfanyl-1,3-dihiole-2-ylium tetrafluoroborate (89.3 mg, 0.378 mmol) in THF-DMF (4:1 = *v*/*v*) at room temperature under nitrogen, and stirring was carried out for 12 h. After separation of the reaction mixture by column chromatography on silica gel (eluent CS_2_) followed by recrystallization from 1:10 CS_2_/hexane, (I) was obtained as black needles in 79% yield.

### Refinement

The H atoms were geometrically positioned with C—H: 0.98 Å, and refined as riding, with *U*_iso_ = 1.5*U*_eq_(C).

The following *SHELX* instructions were applied as restraints during refinement: SADI 0.01 S1C S2C S1A S2A S1B S2B / SADI 0.01 C6C S3C C6A S3A C6B S3B / SADI 0.01 C5C S1C C5A S1A C5B S1B / SADI 0.01 C6C C5C C6A C5A C6B C5B / SADI 0.01 S2C C6C S2A C6A S2B C6B / SADI 0.01 C4C S2C C4A S2A C4B S2B / SADI 0.01 S1C C4C S1A C4A S1B C4B / FLAT S1A S2A S3A C4A C5A C6A / FLAT S1B S2B S3B C4B C5B C6B / FLAT S1C S2C S3C C4C C5C C6C / SIMU 0.01 / ISOR 0.01.

### Crystal data

**Table:**

C_9_H_4_S_7_	*F*(000) = 680
*M**_r_* = 336.54	*D*_x_ = 1.852 Mg m^−^^3^
Monoclinic, *P*2_1_	Mo *K*α radiation, λ = 0.71073 Å
*a* = 17.669 (5) Å	Cell parameters from 794 reflections
*b* = 3.9110 (11) Å	θ = 2.3–23.9°
*c* = 18.380 (5) Å	µ = 1.27 mm^−^^1^
β = 108.177 (4)°	*T* = 93 K
*V* = 1206.8 (6) Å^3^	Needle, black
*Z* = 4	0.09 × 0.02 × 0.02 mm

### Data collection

**Table:**

Bruker APEXII CCD area-detector diffractometer	4975 independent reflections
Radiation source: Bruker TXS fine-focus rotating anode	3396 reflections with *I* > 2σ(*I*)
Bruker Helios multilayer confocal mirror	*R*_int_ = 0.054
Detector resolution: 8.333 pixels mm^-1^	θ_max_ = 27.5°, θ_min_ = 1.9°
phi and ω scans	*h* = −22→15
Absorption correction: multi-scan (*SADABS*; Sheldrick, 1996)	*k* = −5→4
*T*_min_ = 0.894, *T*_max_ = 0.975	*l* = −22→23
6965 measured reflections	

### Refinement

**Table:**

Refinement on *F*^2^	Secondary atom site location: difference Fourier map
Least-squares matrix: full	Hydrogen site location: inferred from neighbouring sites
*R*[*F*^2^ > 2σ(*F*^2^)] = 0.061	H-atom parameters constrained
*wR*(*F*^2^) = 0.107	*w* = 1/[σ^2^(*F*_o_^2^) + (0.0283*P*)^2^] where *P* = (*F*_o_^2^ + 2*F*_c_^2^)/3
*S* = 0.98	(Δ/σ)_max_ = 0.002
4975 reflections	Δρ_max_ = 0.63 e Å^−^^3^
344 parameters	Δρ_min_ = −0.52 e Å^−^^3^
421 restraints	Absolute structure: Flack (1983), 1849 Friedel pairs
Primary atom site location: structure-invariant direct methods	Flack parameter: −0.18 (18)

### Special details

**Table:**

Geometry. All e.s.d.'s (except the e.s.d. in the dihedral angle between two l.s. planes) are estimated using the full covariance matrix. The cell e.s.d.'s are taken into account individually in the estimation of e.s.d.'s in distances, angles and torsion angles; correlations between e.s.d.'s in cell parameters are only used when they are defined by crystal symmetry. An approximate (isotropic) treatment of cell e.s.d.'s is used for estimating e.s.d.'s involving l.s. planes.
Refinement. Refinement of *F*^2^ against ALL reflections. The weighted *R*-factor *wR* and goodness of fit *S* are based on *F*^2^, conventional *R*-factors *R* are based on *F*, with *F* set to zero for negative *F*^2^. The threshold expression of *F*^2^ > σ(*F*^2^) is used only for calculating *R*-factors(gt) *etc*. and is not relevant to the choice of reflections for refinement. *R*-factors based on *F*^2^ are statistically about twice as large as those based on *F*, and *R*– factors based on ALL data will be even larger.

### Fractional atomic coordinates and isotropic or equivalent isotropic displacement parameters (Å^2^)

**Table:**

	*x*	*y*	*z*	*U*_iso_*/*U*_eq_	Occ. (<1)
C4	0.3976 (4)	0.7807 (19)	0.2942 (4)	0.0247 (17)	
C5	0.5339 (4)	1.060 (2)	0.3647 (4)	0.035 (2)	
H5A	0.5870	1.1430	0.3772	0.042*	
C6	0.4937 (4)	1.091 (2)	0.4121 (5)	0.035 (2)	
H6A	0.5160	1.1990	0.4604	0.043*	
C7	0.1148 (4)	0.3154 (18)	0.1591 (4)	0.0190 (16)	
C8	−0.0317 (4)	0.151 (2)	0.1023 (4)	0.0244 (17)	
H8A	−0.0815	0.0767	0.0685	0.029*	
C9	−0.0268 (4)	0.277 (2)	0.1707 (4)	0.0251 (18)	
H9A	−0.0718	0.2915	0.1884	0.030*	
C10	0.3122 (4)	0.6221 (18)	0.8314 (3)	0.0139 (14)	
C11	0.3743 (4)	0.922 (2)	0.9615 (4)	0.0238 (17)	
H11A	0.3839	1.0134	1.0115	0.029*	
C12	0.4298 (4)	0.941 (2)	0.9257 (4)	0.0248 (17)	
H12A	0.4792	1.0526	0.9490	0.030*	
C13	0.1820 (4)	0.1225 (19)	0.5609 (4)	0.0154 (15)	
C14	0.1298 (4)	−0.035 (2)	0.4192 (4)	0.0195 (16)	
H14A	0.0971	−0.1098	0.3703	0.023*	
C15	0.2009 (4)	0.085 (2)	0.4285 (3)	0.0205 (17)	
H15A	0.2218	0.1003	0.3868	0.025*	
C4A	0.3347 (6)	0.633 (2)	0.2437 (4)	0.0190 (17)	0.924 (3)
C5A	0.1947 (5)	0.384 (2)	0.1739 (4)	0.0155 (15)	0.924 (3)
C6A	0.2391 (6)	0.323 (3)	0.1228 (5)	0.021 (2)	0.924 (3)
C4B	0.191 (4)	0.346 (17)	0.181 (4)	0.018 (4)	0.076 (3)
C5B	0.335 (6)	0.59 (2)	0.248 (3)	0.023 (4)	0.076 (3)
C6B	0.331 (2)	0.485 (10)	0.173 (3)	0.023 (3)	0.076 (3)
C4C	0.2640 (3)	0.4578 (17)	0.7717 (3)	0.0170 (15)	
C5C	0.1960 (3)	0.1937 (16)	0.6374 (3)	0.0151 (15)	
C6C	0.1387 (3)	0.1573 (17)	0.6762 (3)	0.0158 (15)	
S4	0.48789 (12)	0.8603 (6)	0.27620 (13)	0.0385 (6)	
S5	0.39615 (10)	0.9263 (6)	0.38449 (10)	0.0270 (5)	
S6	0.05349 (11)	0.1230 (5)	0.07570 (9)	0.0211 (4)	
S7	0.06754 (10)	0.4126 (5)	0.22574 (9)	0.0201 (4)	
S8	0.28575 (11)	0.7290 (5)	0.91400 (10)	0.0219 (5)	
S9	0.40896 (10)	0.7580 (5)	0.83649 (10)	0.0225 (5)	
S10	0.09618 (10)	−0.0554 (5)	0.49847 (9)	0.0189 (4)	
S11	0.25580 (10)	0.2146 (5)	0.51919 (9)	0.0204 (4)	
S1A	0.2450 (5)	0.5721 (17)	0.2628 (3)	0.0191 (11)	0.924 (3)
S2A	0.33680 (13)	0.4760 (6)	0.15541 (12)	0.0277 (6)	0.924 (3)
S3A	0.20601 (13)	0.1459 (6)	0.03754 (11)	0.0275 (6)	0.924 (3)
S1B	0.246 (6)	0.52 (2)	0.268 (3)	0.015 (6)	0.076 (3)
S2B	0.242 (2)	0.312 (11)	0.114 (2)	0.021 (4)	0.076 (3)
S3B	0.4040 (14)	0.512 (7)	0.1345 (13)	0.026 (6)	0.076 (3)
S1C	0.28927 (10)	0.3657 (5)	0.68923 (9)	0.0193 (4)	
S2C	0.16991 (10)	0.3150 (5)	0.76938 (9)	0.0193 (4)	
S3C	0.04904 (10)	−0.0125 (5)	0.64141 (10)	0.0211 (5)	

### Atomic displacement parameters (Å^2^)

**Table:**

	*U*^11^	*U*^22^	*U*^33^	*U*^12^	*U*^13^	*U*^23^
C4	0.025 (3)	0.021 (4)	0.032 (4)	−0.001 (3)	0.014 (3)	0.000 (3)
C5	0.021 (4)	0.028 (5)	0.055 (5)	−0.001 (3)	0.012 (3)	−0.006 (4)
C6	0.024 (4)	0.028 (5)	0.048 (4)	−0.006 (3)	0.002 (3)	−0.004 (4)
C7	0.026 (3)	0.013 (4)	0.017 (3)	0.005 (3)	0.004 (3)	0.000 (3)
C8	0.027 (4)	0.018 (4)	0.022 (3)	−0.004 (3)	−0.001 (3)	−0.001 (3)
C9	0.026 (3)	0.026 (4)	0.025 (4)	0.000 (3)	0.011 (3)	−0.002 (3)
C10	0.016 (3)	0.010 (3)	0.014 (3)	0.002 (3)	0.003 (3)	0.000 (3)
C11	0.028 (4)	0.021 (4)	0.017 (3)	0.003 (3)	−0.001 (3)	−0.002 (3)
C12	0.022 (3)	0.020 (4)	0.022 (3)	−0.002 (3)	−0.009 (3)	0.000 (3)
C13	0.017 (3)	0.010 (3)	0.020 (3)	−0.002 (3)	0.006 (3)	−0.002 (3)
C14	0.020 (3)	0.022 (4)	0.013 (3)	−0.001 (3)	0.000 (3)	−0.001 (3)
C15	0.026 (4)	0.025 (4)	0.012 (3)	0.004 (3)	0.008 (3)	0.003 (3)
C4A	0.020 (3)	0.015 (4)	0.024 (3)	0.000 (3)	0.009 (3)	0.002 (3)
C5A	0.022 (3)	0.015 (3)	0.011 (3)	0.003 (3)	0.008 (3)	0.003 (3)
C6A	0.033 (3)	0.015 (4)	0.014 (4)	0.008 (3)	0.007 (3)	0.000 (3)
C4B	0.025 (5)	0.016 (6)	0.015 (5)	0.004 (5)	0.006 (5)	0.001 (5)
C5B	0.024 (6)	0.020 (6)	0.026 (6)	0.000 (5)	0.010 (5)	0.001 (5)
C6B	0.027 (5)	0.021 (5)	0.024 (5)	0.002 (5)	0.010 (4)	0.001 (5)
C4C	0.012 (3)	0.018 (4)	0.018 (3)	−0.002 (3)	0.001 (3)	−0.003 (3)
C5C	0.012 (3)	0.017 (4)	0.012 (3)	−0.001 (3)	−0.002 (3)	0.000 (3)
C6C	0.019 (3)	0.015 (4)	0.012 (3)	−0.002 (3)	0.004 (3)	−0.001 (3)
S4	0.0305 (11)	0.0339 (16)	0.0588 (14)	−0.0056 (11)	0.0250 (11)	−0.0026 (12)
S5	0.0204 (10)	0.0262 (12)	0.0319 (11)	0.0003 (9)	0.0046 (9)	−0.0031 (10)
S6	0.0292 (11)	0.0190 (11)	0.0142 (9)	−0.0007 (9)	0.0053 (8)	−0.0010 (9)
S7	0.0263 (10)	0.0191 (11)	0.0157 (9)	−0.0017 (9)	0.0075 (8)	−0.0033 (9)
S8	0.0256 (10)	0.0229 (12)	0.0175 (9)	0.0008 (9)	0.0073 (8)	−0.0053 (9)
S9	0.0188 (10)	0.0243 (12)	0.0231 (10)	−0.0012 (9)	0.0047 (8)	−0.0029 (9)
S10	0.0190 (9)	0.0192 (11)	0.0162 (9)	−0.0016 (9)	0.0023 (8)	−0.0002 (9)
S11	0.0201 (10)	0.0231 (12)	0.0176 (9)	−0.0038 (9)	0.0052 (8)	−0.0013 (8)
S1A	0.0212 (12)	0.020 (3)	0.0185 (13)	−0.0022 (18)	0.0095 (12)	−0.0003 (15)
S2A	0.0320 (12)	0.0275 (14)	0.0304 (12)	−0.0002 (11)	0.0197 (10)	−0.0006 (11)
S3A	0.0421 (14)	0.0252 (14)	0.0197 (11)	0.0054 (11)	0.0162 (10)	−0.0023 (10)
S1B	0.015 (7)	0.016 (8)	0.017 (8)	0.000 (7)	0.007 (6)	−0.002 (7)
S2B	0.030 (6)	0.018 (6)	0.016 (6)	0.007 (5)	0.008 (5)	0.004 (5)
S3B	0.027 (8)	0.029 (9)	0.025 (8)	0.009 (7)	0.010 (6)	−0.002 (7)
S1C	0.0158 (9)	0.0238 (12)	0.0174 (9)	−0.0032 (8)	0.0040 (7)	−0.0057 (8)
S2C	0.0171 (9)	0.0224 (12)	0.0178 (9)	0.0004 (8)	0.0047 (8)	−0.0012 (8)
S3C	0.0156 (9)	0.0243 (12)	0.0218 (9)	−0.0010 (8)	0.0035 (8)	0.0004 (9)

### Geometric parameters (Å, °)

**Table:**

C4—C4A	1.336 (11)	C13—C5C	1.378 (8)
C4—C5B	1.38 (9)	C13—S10	1.736 (7)
C4—S4	1.756 (7)	C13—S11	1.744 (6)
C4—S5	1.763 (7)	C14—C15	1.302 (8)
C5—C6	1.292 (9)	C14—S10	1.739 (6)
C5—S4	1.758 (8)	C14—H14A	0.9500
C5—H5A	0.9500	C15—S11	1.723 (7)
C6—S5	1.759 (7)	C15—H15A	0.9500
C6—H6A	0.9500	C4A—S1A	1.743 (6)
C7—C4B	1.28 (6)	C4A—S2A	1.746 (7)
C7—C5A	1.377 (9)	C5A—C6A	1.418 (9)
C7—S7	1.727 (6)	C5A—S1A	1.761 (6)
C7—S6	1.748 (7)	C6A—S3A	1.646 (9)
C8—C9	1.327 (9)	C6A—S2A	1.747 (10)
C8—S6	1.726 (7)	C4B—S2B	1.743 (10)
C8—H8A	0.9500	C4B—S1B	1.745 (10)
C9—S7	1.742 (7)	C5B—C6B	1.417 (11)
C9—H9A	0.9500	C5B—S1B	1.761 (10)
C10—C4C	1.326 (8)	C6B—S3B	1.649 (11)
C10—S9	1.765 (6)	C6B—S2B	1.744 (11)
C10—S8	1.772 (6)	C4C—S2C	1.741 (6)
C11—C12	1.343 (8)	C4C—S1C	1.747 (6)
C11—S8	1.712 (7)	C5C—C6C	1.415 (7)
C11—H11A	0.9500	C5C—S1C	1.760 (6)
C12—S9	1.721 (7)	C6C—S3C	1.652 (6)
C12—H12A	0.9500	C6C—S2C	1.740 (6)
			
C4A—C4—S4	123.6 (5)	C4—C4A—S2A	122.9 (7)
C5B—C4—S4	125 (3)	S1A—C4A—S2A	115.1 (5)
C4A—C4—S5	122.7 (5)	C7—C5A—C6A	125.6 (7)
C5B—C4—S5	121 (3)	C7—C5A—S1A	117.0 (6)
S4—C4—S5	113.6 (4)	C6A—C5A—S1A	117.4 (5)
C6—C5—S4	118.2 (6)	C5A—C6A—S3A	126.5 (8)
C6—C5—H5A	120.9	C5A—C6A—S2A	114.1 (6)
S4—C5—H5A	120.9	S3A—C6A—S2A	119.4 (6)
C5—C6—S5	117.8 (7)	C7—C4B—S2B	120 (5)
C5—C6—H6A	121.1	C7—C4B—S1B	123 (5)
S5—C6—H6A	121.1	S2B—C4B—S1B	114.8 (9)
C4B—C7—S7	117 (3)	C4—C5B—C6B	123 (7)
C5A—C7—S7	120.7 (5)	C4—C5B—S1B	123 (4)
C4B—C7—S6	128 (3)	C6B—C5B—S1B	113 (5)
C5A—C7—S6	125.0 (5)	C5B—C6B—S3B	126 (5)
S7—C7—S6	114.3 (4)	C5B—C6B—S2B	118 (5)
C9—C8—S6	119.1 (6)	S3B—C6B—S2B	116 (3)
C9—C8—H8A	120.4	C10—C4C—S2C	122.5 (4)
S6—C8—H8A	120.4	C10—C4C—S1C	123.1 (4)
C8—C9—S7	115.5 (5)	S2C—C4C—S1C	114.4 (3)
C8—C9—H9A	122.2	C13—C5C—C6C	124.4 (5)
S7—C9—H9A	122.2	C13—C5C—S1C	118.0 (4)
C4C—C10—S9	123.5 (4)	C6C—C5C—S1C	117.5 (4)
C4C—C10—S8	123.4 (5)	C5C—C6C—S3C	126.5 (5)
S9—C10—S8	113.2 (4)	C5C—C6C—S2C	113.8 (4)
C12—C11—S8	117.7 (5)	S3C—C6C—S2C	119.6 (3)
C12—C11—H11A	121.2	C4—S4—C5	95.2 (4)
S8—C11—H11A	121.2	C6—S5—C4	95.2 (4)
C11—C12—S9	118.2 (6)	C8—S6—C7	94.7 (3)
C11—C12—H12A	120.9	C7—S7—C9	96.2 (3)
S9—C12—H12A	120.9	C11—S8—C10	95.6 (3)
C5C—C13—S10	126.7 (5)	C12—S9—C10	95.3 (3)
C5C—C13—S11	119.1 (5)	C13—S10—C14	94.6 (3)
S10—C13—S11	114.3 (4)	C15—S11—C13	95.3 (3)
C15—C14—S10	118.4 (5)	C4A—S1A—C5A	95.6 (3)
C15—C14—H14A	120.8	C6A—S2A—C4A	97.7 (5)
S10—C14—H14A	120.8	C4B—S1B—C5B	98 (2)
C14—C15—S11	117.5 (5)	C6B—S2B—C4B	96 (3)
C14—C15—H15A	121.3	C4C—S1C—C5C	95.7 (3)
S11—C15—H15A	121.3	C6C—S2C—C4C	98.3 (3)
C4—C4A—S1A	122.0 (5)		
